# Do women offered assisted reproduction technologies have a higher incidence of gynecologic cancer? A systematic review and meta-analysis

**DOI:** 10.5935/1518-0557.20170026

**Published:** 2017

**Authors:** Juan Enrique Schwarze, Paulina Valdebenito, Carolina Ortega, Sonia Villa, Javier Crosby, Ricardo Pommer

**Affiliations:** 1 Reproductive Medicine Unit at Clinica Monteblanco; 2 Universidad de Santiago, Chile; 3 Reproductive Medicine Unit at Clinica Las Condes

**Keywords:** Assisted Reproductive Technology, Gynecological cancer, Systematic review

## Abstract

The last two decades have seen an increase in the number of women diagnosed with infertility. The consequent growth in the use of assisted reproductive technologies (ART) calls for the determination of its long-term effects, including the risk of cancer. Many studies have attempted to answer this question, albeit with contradictory results. This review aimed to assess whether assisted reproductive technologies are associated with an increased risk of gynecological cancer. A search for papers in the literature was carried out on MEDLINE, TRIP DATABASE and NICE, resulting in 11 studies enrolling 3,900,231 patients altogether. Of these, 118,320 were offered ART. The incidence of gynecological cancer in the group offered ART was 0.6%, while the incidence in the group not offered ART was 2.1%. Taking all the studies into consideration, women offered ART were not at greater risk of having gynecological cancer; instead, a protective association was found.

## INTRODUCTION

In the last 20 years there has been an increase in the prevalence of infertility and in the use of assisted reproductive technologies (ART). ART, defined as medical procedures involving the ex vivo manipulation of male and female gametes to achieve conception (Luke *et al.*, 2015), have steadily grown in Chile during this period. The number of ART cycles performed in the country has increased by more than 800% -235 to 1932 cases - between 1990 and 2009 (Schwarze *et al.*, 2010). Concerns over the longterm effects of ART have likewise grown.

Since the mid-1960s, there have been reports of an association between the drugs used in ovarian stimulation and several types of gynecologic cancer, particularly ovarian, endometrial and cervical tumors (Siristatidis *et al.*, 2013). Ovarian stimulation is known to expose the ovary to supraphysiological levels of gonadotropins while inducing the development of multiple follicles and a variety of biological effects on the epithelium, together with up to five-fold increases in estradiol blood levels (Zhao *et al.*, 2015). On the other hand, risk factors for cancer often coexist with the characteristics of infertile women (low parity, older age at first birth, early menarche and late menopause, lower incidence and duration of breastfeeding) (Luke *et al.*, 2015). Nevertheless, few studies have looked into the risk of cancer of women undergoing ART, and the evidence establishing possible connections between the disease and ART is little and controversial.

Therefore, the main objective of this review was to assess whether there is an association between assisted reproduction technologies and gynecologic cancer.

## MATERIALS AND METHOD

A search based on keywords "*in vitro* fertilization"; "*in vitro* fertilisation"; "controlled ovarian stimulation"; "Assisted Reproductive Technology"; "IVF" or "ICSI"; "cancer risk"; "ovarian cancer", "endometrial cancer", "cervical cancer", "uterine cancer", "breast cancer" was carried out on NICE, Medline and Trip Database. These keywords were combined using the word AND to generate a subgroup relevant to the search. Studies written in English and Spanish published between June 2000 and June 2016 comparing pregnancies achieved by IVF-TE, ICSI-TE vs. spontaneous conceptions were included. Case reports, case series, meta-analysis, and systematic reviews were excluded. Studies enrolling patients with a history of BRCA1 or BRCA2 mutation, animal studies, studies evaluating fertility preservation in patients diagnosed with cancer, and studies referring to other types of cancer were excluded.

The papers were selected based on their titles and abstracts. The references cited in each of the papers were reviewed, and papers deemed relevant were added to our review. Two authors (JES and PV) reviewed these papers to check whether they met the inclusion and exclusion criteria. Disagreements between the authors were settled either by group discussions or by a third reviewer.

Statistical package Stata (Statacorp, USA) was used to treat the meta-analysis data. Heterogeneity between studies was assessed by the chi-square test. A fixed-effect model was used for the meta-analysis and odds ratios (OR) were calculated with a 95% confidence interval (95% CI) using the Mantel-Haensze test. The results were represented in a forest plot.

## RESULTS

**Figure 1:**
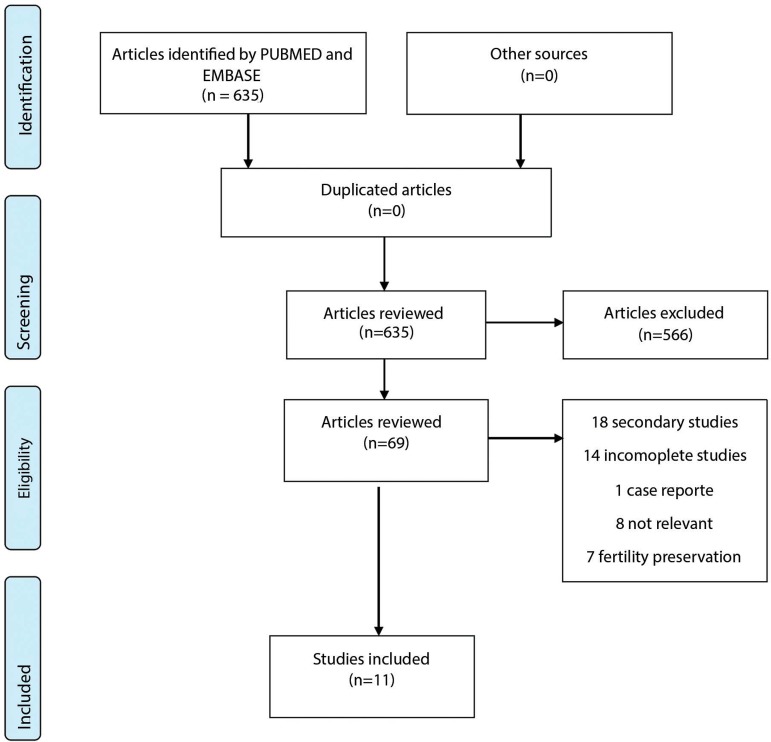
Selection of papers for systematic review.

The first search produced 69 eligible papers; however, only 11 met the inclusion criteria and had none of the exclusion criteria (Fig. 1). Seven of the 11 papers reviewed were produced in Western Europe. The oldest study included was published in 2006 (Kristiannson *et al.*, 2007) and the most recent in 2015 (Reigstad *et al.*, 2015). The number of women with gynecologic cancer in the ART group ranged from 11 to 148, with a total of 714 cases. In the control group, the number of patients with gynecologic cancer ranged from 13 to 48,619, with a total of 79,610 patients. In most of the papers, the data was adjusted for maternal age, age at first birth, and parity.

The main outcome observed was gynecologic cancer after ART procedures. Three of the 11 papers included in our review looked into the overall risk of different types of cancer, including gynecologic tumors; three assessed the risk of ovarian cancer and two assessed exclusively the risk of gynecologic cancer subsequent to ART procedures. Table 1 shows a summary of the included papers.

**Table 1:** Summary of articles included

Author	Methodology	Principal findings
Kristiansson *et al*., 2007	Prospective cohort analysis performed in Sweden between 1981-2001	No increase in the risk of developing postmenopausal cancer in women with a delivery after IVF compared with controls
Sanner *et al*., 2009	Retrospective cohort analysis of 2,768 women with infertility treatment between 19761-1975. The main comparison was the use of gonadotropins and clomiphene citrate.	Five-fold increase in the risk of cancer in women who took gonadotropins
Källén *et al*., 2011	Retrospective cohort analysis of women with delivery after IVF between 1982-2006. Cases of gynecologic cancer were found by cross-referencing the patients in the cohort against the Cancer Database.	Low risk of breast and cervical cancer.No change in the risk of other types of cancer.
van Leeuwen *et al*., 2011	Retrospective cohort analysis	Data suggest an increase of cancer after controlled ovarian hyperstimulation
Yil-Kuha *et al*., 2012	Retrospective cohort analysis of 18,350 Finnish women treated between 1996-1998. Cancer cases were identified from a cancer database	Three-fold increase in the risk of ovarian cancer in women offered IVF.
Stewart *et al*., 2012	Retrospective cohort analysis performed in Australia between 1982-2002	Increased risk of breast cancer in women offered ART at younger ages
Stewart *et al*., 2013	Retrospective cohort analysis of 21,639 Australian patients diagnosed with infertility or offered infertility treatment; the individuals were cross-referenced to a cancer database	Women with a history of ART are at higher risk of having borderline ovarian tumors.
Stewart *et al*., 2013	Retrospective cohort analysis in Australia, between 1982-2002	No evidence of increased risk of ovarian cancer after post-IVF delivery.
Reigstad *et al*., 2015	Retrospective cohort analysis of 808,834 Norwegian women after delivery, linked to the national cancer database. (1984-2010)	Increased risk of breast cancer in women with post-ART deliveries versus women with spontaneous conception delivery.
Reigstad *et al*., 2015	Retrospective cohort analysis of 806, 248 women registered in the Norway Birth Register between 1984 and 2010. Gynecologic tumors were identified by cross-referencing the enrolled individuals to the Cancer Database	Increased risk of cancer after ART; however, after correction for confounding factors, the difference was not significant.
Kessous *et al*., 2016	Retrospective cohort study of 106,031 Israeli women with a history of either IVF or ovulation induction.	Increased risk of gynecologic cancer in women with history of IVF

In a prospective cohort study carried out in Sweden from 1981 to 2001, Kristiansson *et al.* (2007) assessed the risk of invasive or *in situ* gynecologic tumors after ART procedures, and compared women offered IVF to women without a history of infertility. The patients in the IVF group were followed for 6.2 years and the individuals in the control group were followed for 7.8 years. Premenopausal women who had their babies after IVF had little or no increase in the risk of developing cancer, and lower incidences of cervical or ductal carcinoma *in situ* were observed after ART procedures (odds ratio: 0.570; 95% confidence interval: 0.503-0.646).

Sanner *et al.* (2009) performed a retrospective cohort study, in which a total of 2.768 women treated for infertility and/or disorders associated with infertility between 1961 and 1975 were evaluated. Patients exposed to clomiphene citrate and/or gonadotropins were analyzed. The mean follow-up period was 33 years. The results revealed an association between exposure to gonadotropins and a 5-6-fold increase in the risk of developing cancer (OR: 1.873, 95% CI: 0.793-4.427).

Källén *et al.* (2011) carried out a retrospective cohort study in Sweden from 1982 to 2006 to assess the global risk of cancer including gynecologic tumors. The authors reviewed cases of women who had their babies after IVF *versus* women who delivered their babies after spontaneous conception, and found low risk of breast or cervical cancer in the IVF group (OR: 0.369; 95% CI: 0.344-0.396).

van Leeuwen *et al.* (2011) published a retrospective cohort study in which 25,152 women with fertility issues were assessed; some were given IVF. The authors found that ovarian stimulation for IVF may increase the risk of malignant ovarian tumors, especially of the borderline type (OR: 1.187; 95% CI: 0.702-2.005).

In Finland, Yli-Kuha *et al.* (2012) analyzed the cases of 18,350 patients between 1996 and 1998, and found that the incidence of ovarian cancer was three times higher in the group offered IVF when compared to the control group. The incidence of borderline tumors was similar in both groups (OR: 0.778; 95% CI: 0.604-1.002).

Stewart *et al.* (2012) conducted a retrospective cohort study to assess the occurrence of breast cancer in women receiving IVF treatment. The authors found an increase in breast cancer rates in women of earlier ages receiving IVF, but were unable to find a positive association between breast cancer and late use of IVF (OR: 1.166; 95% CI: 0.944-1.441).

Stewart *et al.* (2013) analyzed 21,639 charts of patients diagnosed with infertility or referred to procreative management linked to the national cancer registry. Women submitted to IVF were at increased risk of having borderline ovarian tumors (OR: 1.376; 95% CI: 0.706-2.683). The same author (Stewart *et al.*, 2013) conducted a third study using the same cohort of patients, but failed to find increased risk of ovarian cancer in the group given IVF (OR: 2.413; 95% CI: 1.152-5.054).

Reigstad *et al.* (2015) analyzed the data of 808,834 women included in the Norwegian Birth Registry also linked to the Norwegian Cancer Registry between 1984 and 2010. The authors described increased risk of breast cancer for women who gave birth after ART procedures when compared to women with spontaneously conceived babies (OR: 0.844; 95% CI: 0.723-0.985). In 2015, Reigstad *et al.* (2015) studied the cases of 806,248 women and found increased risk of cancer in general; however, such increase was not significant after correction for multiple analyses (OR: 0.697; 95% CI: 0.541-0.897).

Kessous *et al.* (2016) looked into the cases of 106,031 patients offered IVF, with OI and without a diagnosis of infertility, seen in Israel between 1988 and 2013. Patients with a history of IVF were at higher risk of having ovarian or uterine cancer when compared to patients with OI and patients without a history of infertility (OR: 2.181; 95% CI: 1.130-4.208).

The combined odds ratio of the studies for risk of gynecologic cancer in patients given IVF versus the risk of unexposed patients was 0.519 (95% CI: 0.493-0.547)

## DISCUSSION

**Figure 2:**
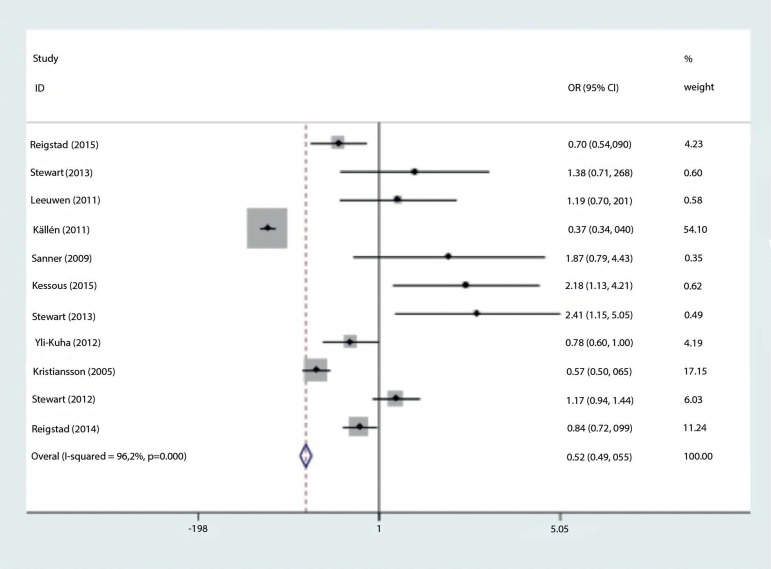
Forrest plot analysis.

The results of the present study do not support the idea that ART procedures increase the risk of gynecologic cancer; instead, the data suggest a protective association (Figure 2 Forest Plot), as also described in previous systematic reviews. Most were unable to find a relationship between ART and ovarian, endometrial or breast cancer (Impicciatore & Tiboni, 2011; Li *et al.*, 2013; Sergentanis *et al.*, 2014; Zhao *et al.*, 2015).

The primary strengths of this review are the large number of patients from both groups and the fact that they reflect populations from different countries, which allows for generalization of results. The limitations revolve around the fact that not all studies contained detailed information on the drugs used in ART protocols, duration of treatment, number of cycles, or dosages. The cohorts were not equivalent, since in some studies the control groups were infertile patients, while in others controls were fertile.

The short follow-up period and the young age of the patients enrolled in the studies might explain the small number of cases of gynecologic cancer reported. Only three studies - Sanner *et al.* (2009), van Leeuwen *et al.* (2011) and Kessous *et al.* (2016) - followed patients offered ART procedures for more than ten years. A long follow-up period is required, particularly if one considers that the incidence of ovarian and endometrial cancer increases after menopause, while ART procedures are mostly performed during the last reproductive years.

Another confounding factor is the lack of accurate information regarding family history of cancer, age of menarche, age at first delivery, parity, use of oral contraceptives or hormone replacement therapy, and BMI.

Despite the limitations of this review, the implications arising from its findings are rather reassuring from the standpoint of public health, since they support the results of earlier reviews on the effects of ART on the onset of gynecologic cancer. However, further cohort studies are needed to examine infertile women receiving ART procedures *versus* infertile women not offered ART procedures, adjusting the findings for age at the beginning of treatment, drug protocol, number of stimulation cycles, BMI, and family history of cancer to determine the effects of assisted reproductive technologies on gynecologic cancer. In addition, longer follow-up periods are required to determine with greater certainty the long-term effects of ART procedures considering the longer life expectancy observed in women globally.

ART procedures are still a novelty in medicine. Therefore, the long-term effects associated with ART are yet to be determined. Large international multicenter cohort studies are required to find whether there is risk associated with ART procedures, so that patients are ultimately better advised and followed up.
